# Virtuelle Flurgespräche und Status-Updates: Zusammenarbeit in avatar-basierten Arbeitsumgebungen

**DOI:** 10.1007/s41449-023-00356-8

**Published:** 2023-03-21

**Authors:** Felix Oehring, Markus A. Feufel, Frauke Mörike

**Affiliations:** Institut für Psychologie und Arbeitswissenschaft (IPA), Fachgebiet Arbeitswissenschaft, Technische Universität Berlin, Straße des 17 Juni 135, 10623 Berlin, Deutschland

**Keywords:** Virtualität, Hybrides Arbeiten, Kollaboration, Avatar-basierte Arbeitsumgebungen, Virtuality, Hybrid work, Collaboration, Avatar-based work environments

## Abstract

Der vorliegende Beitrag beschäftigt sich mit der Zusammenarbeit von hybrid arbeitenden Teams in avatar-basierten virtuellen Büroumgebungen. Ausgehend von den drei Dimensionen der Virtualität gehen wir folgenden Forschungsfragen nach: (1) Wie wird der Arbeitsalltag und die Zusammenarbeit in diesen Umgebungen koordiniert? und (2) Welche Vorteile und Herausforderungen sehen Nutzer*innen in Bezug auf avatar-basierte Arbeitsumgebungen? Auf Basis einer multi-methodischen Studie bestehend aus qualitativen Interviews mit erfahrenen Nutzer*innen und einer partizipativen Fokusgruppendiskussion mit neuen Nutzer*innen zeigen wir, dass Zusammenarbeit in avatar-basierten Arbeitsumgebungen in vielfältigen Arbeitsformen – von Ko-Präsenz bis zum mobilem Arbeiten – stattfindet und dass für die Koordination dieser Arbeitsmodi vielversprechende Ansätze zur Verfügung stehen. Unsere Ergebnisse zeigen aber auch, dass für die Ausschöpfung dieses Potentials nicht nur die virtuellen Umgebungen selbst, sondern auch die kollaborativen Arbeitsformen und die digitale Infrastruktur von Teams konsequent weiterentwickelt werden müssen.

*Praktische Relevanz*: Die Studie untersucht Virtualität anhand einer aktuell nutzerbaren, avatar-basierten Arbeitsumgebung und zeigt die Perspektiven erfahrener und neuer Nutzer*innen auf Zusammenarbeit innerhalb dieses soziotechnischen Arbeitssystems auf. Insbesondere werden konkrete kollaborative Arbeitspraktiken vorgestellt und deren Herausforderungen diskutiert, um potenziellen Nutzer*innen, die virtuelle Arbeitsumgebungen selbst anwenden möchten, eine Orientierung zu bieten.

## Abstract (en)

This article focuses on the collaboration of hybrid teams in avatar-based virtual office environments. Based on the three dimensions of virtuality, we pursue the following research questions: (1) How is everyday work and collaboration coordinated in these environments? and (2) Which advantages and challenges do users perceive in relation to this form of work? Based on a multi-method study consisting of qualitative interviews with experienced users and a participatory focus group discussion with new users, we illustrate that collaboration in avatar-based work environments is marked by a diverse array of work practices—ranging from co-present to mobile work—and that promising implementation methods exist to coordinate these practices. However, our results also suggest that to exploit this potential, not only the virtual environments but also teams’ work practices and digital infrastructure must be further developed.

*Practical relevance*: The study discusses virtuality based on a currently used avatar-based work environment and illustrates the perspectives of experienced and new users on collaboration in these socio-technical work systems. Specifically, we present concrete implementations and challenges of collaborative work practices in these virtual environments, which offer orientation for practitioners interested in applying these solutions to their own work contexts.

## Einführung

Die Welt verändert sich gerade jetzt. […] Ich glaube nicht, dass es möglich ist, ein Unternehmen zu haben, in dem alle immer vor Ort sind. (Interview E4)

Die COVID-19 Pandemie und der damit verbundene Lockdown haben die potenziellen Vorteile und Möglichkeiten virtueller Zusammenarbeit aufgezeigt, aber ebenso die Herausforderungen sichtbar gemacht, die digital mediierte Arbeitsformen mit sich bringen: Durch den Wegfall informeller Flurgespräche wurden arbeitsrelevante Netzwerke geschwächt und in der Konsequenz nahm nicht nur der synchron, sondern auch der asynchron über digitale Medien vermittelte Informationsaustausch zu (Yang et al. 2022). Für die Zukunft wird davon ausgegangen, dass mobil und hybrid arbeitende Teams den Arbeitsalltag vieler Menschen weiterhin prägen werden (Nelson et al. 2022). Aber wie sollten effektive Arbeitspraktiken für die hybride Zusammenarbeit aussehen bzw. gestaltet werden? Dieser Artikel bietet einen ersten Eindruck von kollaborativen Arbeitspraktiken in hybrid arbeitenden Teams und trägt damit zu den dringend benötigten empirischen Befunden über diese neu entstehenden Arbeitsformen bei. Dafür werden virtuelle Arbeitsumgebungen in den Blick genommen, in denen Team-Mitglieder durch eine virtuelle Kunstfigur – einen Avatar – repräsentiert sind. Die Anbieter solcher avatar-basierter Arbeitsumgebungen versprechen effektive Zusammenarbeit in verteilt oder hybrid arbeitenden Teams, wenn physische Ko-Präsenz aller Mitglieder nicht gegeben ist. Auch wenn bereits vor 2020 Ansätze aus dem Kontext der Multi-Player Games und virtualisierter Büroräume existierten (z. B. Honda et al. 1997), sind sie erst durch die Pandemie wirklich bekannt bzw. auf breiterer Ebene umgesetzt worden (Silva et al. 2022). Seit 2020 sind zunehmend Anbieter avatar-basierter Arbeitsumgebungen auf dem Markt, die sowohl zeitlich begrenzte virtuelle Orte für Konferenzen (z. B. Laval Virtual Worlds) als auch auf langfristige Nutzung angelegte, virtuelle Büroumgebungen anbieten (z. B. WorkAdventure oder Gather.Town).

**Figure 1:**
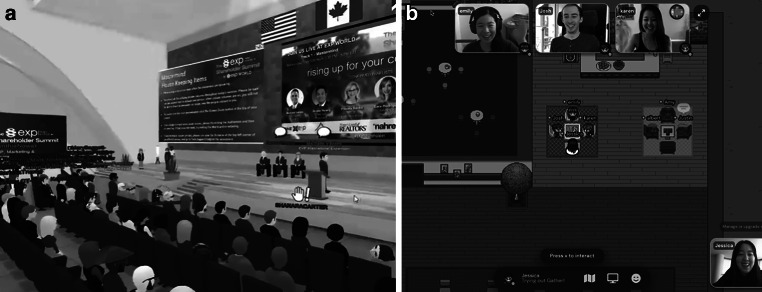

Allen diesen Anwendungen ist gemeinsam, dass sich die Nutzer*innen innerhalb der virtuellen Umgebungen mit einem selbst gestaltbaren Avatar frei bewegen können, um den Arbeitsalltag in einem physischen Büro bzw. auf einer Konferenz zu ersetzen bzw. zu ergänzen (Abb. 1). In Bezug auf die Arbeit in und mit diesen virtuellen, avatar-basierten Büroumgebungen gehen wir in dieser Studie anhand des Konzepts der Virtualität (Kirkman und Mathieu 2005) zwei Forschungsfragen nach: (1) Wie wird der Arbeitsalltag und die Zusammenarbeit in diesen Umgebungen koordiniert? und (2) Welche Vorteile oder Herausforderungen sehen erfahrene und neue Nutzer*innen in Bezug auf avatar-basierte Arbeitsumgebungen? Insbesondere steht dabei die Zusammenarbeit in hybrid arbeitenden Teams im Vordergrund, bei denen ein Teil der Mitarbeitenden physisch im Büro sitzt und mehrere Kolleg*innen mobil bzw. verteilt arbeiten.

Im Folgenden wird zunächst auf den Stand der Forschung eingegangen (Kap. 2) und anschließend das Studiendesign vorgestellt (Kap. 3), bevor die Ergebnisse anhand besonders anschaulicher Originalzitate präsentiert (Kap. 4) und zusammen mit den daraus ableitbaren Arbeitsformen diskutiert werden (Kap. 5). Unser Fazit wird mit einem Ausblick auf zukünftige Forschungsbedarfe verbunden (Kap. 6).

## Stand der Forschung

### Virtualität als mehrdimensionales Konstrukt

Ein definierender Faktor von hybriden Teams ist, dass einige Kolleg*innen in Ko-Präsenz an einem gemeinsamen Arbeitsplatz zusammenarbeiten und weitere Teammitglieder sich an einem oder mehreren anderen Orten befinden, mit denen virtuell kommuniziert und gearbeitet wird (Griffith und Neale 2001; Busboom 2023). In den meisten Studien zu hybriden wie virtuellen Arbeitsweisen steht genau dieser Aspekt, d. h. die geografische Dispersion bzw. räumliche Trennung, im Vordergrund (Al-Ani et al. 2012; Garrison et al. 2010; Jarvenpaa und Leidner 1999; Maznevski und Chudoba 2000; Yang et al. 2022). Darüber hinaus zeigt die systematische Literatur Review von Clark et al. (2019), dass eine Vielzahl von Studien die Verwendung von Informations- und Kommunikationstechnologien (IKT) als weiteres definierendes Element von virtueller Arbeit fokussiert (Beata Krawczyk-Bryłka 2017; Neumayr et al. 2022; Neves de Souza et al. 2023). Mit diesen ein- bzw. zweidimensionalen Modellen von Virtualität werden jedoch meist nur die dichotome Unterscheidung zwischen virtueller und face-to-face Arbeit (z. B. in Bezug auf die Arbeitszufriedenheit) analysiert (Martins et al. 2004). Mögliche Zwischenformen oder hybride Arbeitsstrukturen werden außen vorgelassen. Eine ein- oder auch zweidimensionale Perspektive ist daher für eine nuancierte Betrachtung virtueller und hybrider Zusammenarbeit sowie der Identifikation neuer Gestaltungsmöglichkeiten dieser Arbeitsformen nicht ausreichend. In hybriden Strukturen arbeiten virtuellen Teams beispielsweise nicht nur von verteilten Standorten aus, sondern kommunizieren, auch wenn sie am gleichen Standort arbeiten, weiter über technische Hilfsmittel. Um das Kontinuum virtueller Arbeitsformen untersuchen zu können, sollte daher zumindest eine dritte Dimension ergänzt werden, die erfasst, welche virtuellen Medien wann, zu welchem Zweck und wie häufig als Kommunikations- und Kooperationswerkzeug verwendet werden (Kauffeld et al. 2016; Neves de Souza et al. 2023).

Für den theoretischen Rahmen dieser Studie verwenden wir die dreidimensionale Ausprägung von Virtualität nach Kirkman und Mathieu (2005), da es nicht nur eines der ersten Modelle ist, das Virtualität als mehrdimensionales Konstrukt erfasst, sondern bereits in zahlreichen Studien zur Virtualität erfolgreich eingesetzt wurde (z. B. Kauffeld et al. 2016; Mesmer-Magnus et al. 2011; Ortiz de Guinea et al. 2012; Hill und Villamor 2022; Schmutz et al. 2023). Das Modell nimmt dabei folgende drei Dimensionen virtueller Arbeit in den Blick: (1) die Art und Menge an Informations- und Kommunikationstechnologien (IKT), (2) die Art und Menge an Informationen, die über diese IKT ausgetauscht werden (d. h. den Informationsgehalt) und (3) die räumliche und zeitliche Synchronität der Arbeitsprozesse. Im Folgenden umreißen wir die einzelnen Dimensionen knapp und ordnen sie in den Forschungsstand ein:

1. **Verwendung von Informations- und Kommunikationstechnologien. **Durch die rasante Digitalisierung in den letzten Jahrzehnten werden immer mehr digitale Technologien in den Arbeitsalltag von Teams integriert (Meluso et al. 2022). Um zwischen reinen face-to-face Teams, hybriden Teams und ausschließlich virtuellen Teams zu unterscheiden, beschreiben Griffith und Neale (2001) daher einen linearen Zusammenhang zwischen dem Umfang und der Häufigkeit, mit der digitale Werkzeuge verwendet werden, und der Virtualität eines Teams. Das bedeutet, die Virtualität steigt, je mehr bzw. je häufiger Technologien verwendet werden wie Instant Messengers, Blogs, Kalenderplattformen, online-basierte Projektmanagementsysteme, E‑Mails, Videokonferenzsysteme und computerbasierte Überwachungssysteme (Ganesh und Gupta 2010; Meluso et al. 2022; Vocke und Mörike 2020). Mithilfe dieser Dimension betrachten wir in dieser Studie das Zusammenspiel unterschiedlicher IKT-Lösungen in avatar-basierten Büroumgebungen.
2. **Informationsgehalt der Medien.** Die zweite Dimension der Virtualität beschreibt die Art und Menge an Informationen, die über ICTs ausgetauscht werden können. Basierend auf der Media Richness Theory kann der Informationsgehalt eines Mediums definiert werden als „… the ability of information to change understanding within a time interval“ (Daft und Lengel 1986, S. 560). Dabei sei Information umso gehaltvoller, je mehr persönliche Nuancen mitkommuniziert werden können und je besser direktes Feedback gegeben werden kann, um Zweideutigkeiten und Unsicherheiten im Austausch zu vermeiden. Während die reine face-to-face Kommunikation als reichhaltigste Austauschform eingestuft wird (Morrison-Smith und Ruiz 2020; Palmer und Speier 1998), ist die E‑Mail als ein Medium mit weniger Informationsreichtum zu klassifizieren. Sie kann nur Text übertragen, ermöglicht lediglich verzögerte Rückmeldung/-fragen und eignet sich demnach weniger für die Übermittlung mehrdeutiger, komplexer Informationen (z. B. persönliche Konflikte). Dagegen können E‑Mails effektiv genutzt werden, um eindeutige, einfache Mitteilungen, wie zum Beispiel eine Terminvereinbarung, auszutauschen (Kauffeld et al. 2016; Kirkman und Mathieu 2005). Der bedarfsgerechte Einsatz reichhaltigerer Kommunikationsmittel in digitalen Arbeitsumgebungen ermöglicht eine höhere Virtualität (Ebrahim et al. 2009) und hat wesentlichen Einfluss auf die Teameffektivität (Daft und Lengel 1986). In dieser Arbeit wird diese Dimension eingesetzt, um den Informationsgehalt von Avataren in den virtuellen Büroumgebungen zu erfassen.
3. **Synchronität.** Die dritte Dimension von Virtualität, die von Kirkman und Mathieu (2005) definiert wurde, ist die Synchronität, wobei die Autoren von zwei Extremen ausgehen: der synchronen und der asynchronen Zusammenarbeit. Der Vorteil der synchronen Zusammenarbeit liegt vorwiegend in dem direkten und unmittelbaren Austausch von Informationen und der Diskussion, wohingegen asynchrone Kommunikation effektiv genutzt werden kann, um zum Beispiel Hintergrundinformationen für eine Diskussion zur Verfügung zu stellen (Kirkman und Mathieu 2005). Ausgehend von der Media Richness Theory (Daft und Lengel 1986) verknüpfen Dennis et al. (2008) den effektiven Einsatz (a-)synchroner Medien mit zwei Zielsetzungen von Kommunikation – der Konvergenz des Verständnisses und der Informationsübermittlung. Um Informationen erfolgreich zu übermitteln, ist ein geringeres Niveau an medialer Synchronität notwendig, da Informationen auch retrospektiv angeschaut und verarbeitet werden können. Hingegen entsteht ein gemeinsames Verständnis (Konvergenz) eher, wenn synchrone Medien genutzt werden. Für avatar-basierte virtuelle Büroumgebungen birgt diese Dimension eine analytische Linse, um nach spezifischen Zielsetzungen der Kommunikation zu fragen.

Diese drei Dimensionen von Virtualität werden im Folgenden als Leitdimensionen für die Analyse und Darstellung der Ergebnisse dienen. Sie werden aber zunächst um den aktuellen Stand der Forschung zu avatar-basierten Arbeitsumgebungen ergänzt.

### Avatar-basierte Arbeitsumgebungen

**Figure 2:**
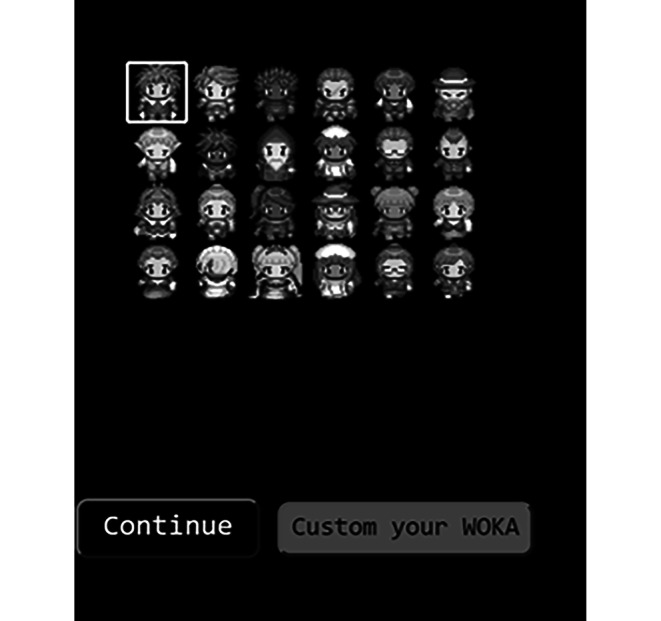

Während das Konzept des Avatars bereits seit langem und erfolgreich im Bereich der unterhaltungsorientierten Videospiele und Serious Games mit mehreren Spielern eingesetzt wird (Oksanen et al. 2013; Hudson und Hurter 2016), sind Avatare in virtuellen Arbeitsumgebungen ein neueres Phänomen. Bisher wurde die Einbettung von Avataren in einen simulierten Büro- und Arbeitskontext zum Beispiel verwendet, um auf genderbedingte Voreingenommenheit hinzuweisen (Beltran et al. 2021). Avatar-basierte Arbeitsumgebungen werden in diesem Beitrag als virtuelle Plattformen definiert, die Videokonferenzsysteme in eine flexibel gestaltbare Umgebung einbinden (Albers-Heinemann 2020). Die Mitarbeiter*innen treten der Plattform als Avatare bei, d. h. als eine computergenerierte Figur, die von dem/der Nutzer*in verwendet wird, um mit weiteren Avataren und Objekten in der virtuellen Arbeitsumgebung zu interagieren (siehe Abb. 2). Die individuell ausgestalteten Avatare können sich in der virtuellen Umgebung frei bewegen, was den dort verfügbaren Raum erlebbar macht (Rings 2021).

Sobald eine Person den virtuellen Raum betritt, kann diese mit den Menschen, die sich in dieselbe Umgebung eingeloggt haben, über die Avatare kommunizieren. Ein wesentlicher Vorteil avatar-basierter Arbeitsumgebungen besteht darin, dass jederzeit und ohne großen Aufwand informelle Einzel- oder Gruppengespräche begonnen werden können (Albers-Heinemann 2020). Für einen kurzen persönlichen Austausch in der „virtuellen Kaffeeküche“ gehen die Avatare innerhalb des virtuellen Raumes einfach aufeinander zu und es öffnet sich automatisch der Gesprächsbereich eines Videokonferenztools. Diese Funktionalität ist ein wesentlicher Unterschied zur herkömmlichen terminierten Einbindung von Videokonferenzsystemen in die Arbeitspraxis und soll den sozialen Kontakt und den Aufbau von interpersonellen Beziehungen auch in virtuellen Settings ermöglichen (Rings 2021; Schwär 2020). Ähnliche Befunde zeigten sich auch für die Online-Lehre im Hochschulbereich (Latulipe und De Jaeger 2022; Silva et al. 2022). Indem avatar-basierte Arbeitsumgebungen auf diese Weise die face-to-face Zusammenarbeit virtuell nachbilden, bieten sich diese Umgebungen an, den Informationsgehalt und die Synchronität virtueller Zusammenarbeit in Zukunft noch weiter steigern (Schwär 2020). Allerdings legen die Befunde für die Nutzung von Avataren im Kontext sozial motivierter Interaktion nahe, dass bei der Gestaltung von Avataren noch weitere Forschung notwendig ist, um Gespräche über sensible und persönliche Themen (Moustafa und Steed 2018) sowie kreative Zusammenarbeit (de Rooij et al. 2017) zu unterstützen.

## Methoden

### Datenerhebung

Um einen ersten Eindruck der gelebten Arbeitspraxis sowie der Erfahrungen von Studienteilnehmer*innen mit avatar-basierten Arbeitsumgebungen zu gewinnen, wurde ein zweiteiliges, qualitatives Forschungsdesign entwickelt, das (1) fünf halbstrukturierte Interviews mit erfahrenen Nutzer*innen virtueller Büroumgebungen umfasst sowie (2) eine interaktive Fokusgruppendiskussion mit Teilnehmer*innen ohne Vorerfahrung, die gebeten werden, zum ersten Mal in einer virtuellen Büroumgebung zusammen zu arbeiten und über dieser Erfahrung zu reflektieren. Da zum Zeitpunkt der Erhebung im Mai bis Juli 2021 kaum empirische Befunde zugänglich waren, wurde hier ganz bewusst eine Forschungsstrecke mit explorativem Charakter gewählt, um sowohl über die tatsächliche Arbeitspraxis in virtuellen Arbeitsumgebungen sprechen zu können als auch explorative und hypothetische Überlegungen der Teilnehmer*innen mit aufnehmen zu können. Die Datenerhebung erfolgte ausschließlich virtuell.

Als avatar-basierte Arbeitsumgebung wurde die Lösung von WorkAdventure (workadventu.re) gewählt, auch wenn es weitere Anbieter auf dem Markt gibt (z. B. GatherTown und Wonder.Me), deren Funktionalitäten dem Angebot von WorkAdventure ähnlich sind. Die Fokussierung auf eine spezifische Lösung schafft einen konstanten Kontext, um die Einschätzungen der Nutzer*innen vergleichen und aufeinander beziehen zu können. Zum anderen kamen forschungspraktische Faktoren hinzu: vom 27. bis 30. Dezember 2020 wurde die jährliche Konferenz des ChaosComputerClubs mit mehreren tausend Teilnehmer*innen erstmals als virtuelle Tagung mit der Lösung von WorkAdventure durchgeführt (R3C 2020). Nicht zuletzt wurde dadurch diese Lösung als OpenSource-Angebot unter IT-Expert*innen im deutschsprachigen Raum bekannt.

Für den ersten Teil der Studie, eine Interviewstrecke mit fünf halbstrukturierten Interviews von jeweils ca. 60 min, wurde ein Interviewleitfaden auf Basis einer Literaturrecherche und einer Analyse der aktuell im Markt verfügbaren avatar-basierten Arbeitsumgebungen erstellt. Die Fragenbereiche orientierten sich an den Dimensionen der Virtualität und bezogen sich auf Aspekte der Zusammenarbeit in einem hybrid arbeitenden Team sowie konkrete Rückmeldungen zur Gestaltung der genutzten avatar-basierten Plattform. Dabei wurde dezidiert nach positiven und negativen Wahrnehmungen und Erfahrungen mit der virtuellen Büroumgebung gefragt. Der eingesetzte Interviewleitfaden diente vor allem der Orientierung innerhalb der Interviews, d. h. es bestand die Möglichkeit von dem Leitfaden abzuweichen, um spezifische Nachfragen zu stellen (Przyborski und Wohlrab-Sahr 2021). Die ersten Versionen des Leitfadens wurden iterativ in drei Pilotinterviews erprobt und auf Basis der Ergebnisse angepasst (ein Auszug des finalen Leitfadens ist im Anhang zu finden).

Die fünf erfahrenen Interviewpartner*innen waren entweder Entwickler*innen der avatar-basierten Plattform oder erfahrene Nutzer*innen, die die virtuelle Plattform zwischen 3 Monaten bis 1,5 Jahren in unterschiedlichen Rollen als Alltagsnutzer*innen oder Systemadministrator*innen der Plattform aktiv genutzt haben. Für die Rekrutierung wurden Webseiten von Unternehmen auf Updates, Blogbeiträge oder Tweets überprüft, die über die Nutzung von avatar-basierten Büroumgebungen berichteten. Diese Unternehmen wurden gezielt mit einer Interviewanfrage angeschrieben. Parallel dazu wurden auch die unterschiedlichen Anbieter der virtuellen Plattformen kontaktiert und um Interviews mit Mitgliedern des Entwicklungsteams gebeten. Eine Übersicht der Interviewpartner*innen inkl. ihrer Arbeitserfahrung in avatar-basierten virtuellen Büroumgebungen finden sich in Tab. 1.

**Table 1:** 

	Nutzungsprofil d. Interviewpartner*in	Erfahrung	Interviewdauer
E1	Systemadministrator*in	5 Monate	62 min
E2	Alltagsnutzer*in	4 Monate	56 min
E3	Systemadministrator*in	3 Monate	62 min
E4	Entwickler*in	1,5 Jahre	65 min
E5	Alltagsnutzer*in	11 Monate	70 min

Der zweite Teil der Studie wurde als eine interaktive Fokusgruppendiskussion (Dauer: 60 min) mit 5 Personen durchgeführt, die bislang keine Erfahrung mit avatar-basierten Arbeitsumgebungen hatten. Hierfür konnte ein Team eines internationalen Logistikunternehmens rekrutiert werden, deren Mitglieder sich selbst als grundsätzlich technikaffin und an neuen Arbeitstechnologien interessiert eingestuft haben. Die Fokusgruppendiskussion fand in einer virtuellen Büroumgebung statt, in der sich die Teilnehmer*innen zunächst einen eigenen Avatar erstellen und damit frei in der Arbeitsumgebung bewegen konnten, um erste Erfahrungen zu sammeln. Diese „konkrete soziale Situation“ (Przyborski und Wohlrab-Sahr 2021, S. 173) war ein essenzieller Faktor für die erfolgreiche Durchführung dieser Fokusgruppendiskussion, denn sie wurde genutzt, um im Verlauf der Diskussion über die spezifischen Eindrücke des Teams und deren Wahrnehmung über den Eintritt in die avatar-basierte Plattform zu diskutieren. Der Erstautor dieses Beitrags (FO) fungierte als Diskussionsleiter, der nach der oben beschriebenen Orientierungsphase alle Teilnehmer*innen für die Fokusgruppendiskussion in den Konferenzraum des virtuellen Büros einlud. Auch für die Fokusgruppendiskussion wurde ein Leitfaden verwendet, um zunächst die Eindrücke strukturiert zu erfassen, bevor die Diskussion in einen freien Austausch der Teilnehmer*innen untereinander überging.

### Datenauswertung

Alle Interviews wurden aufgezeichnet und nach der Methode des einfachen Transkriptionssystems von Dresing und Pehl (2015) transkribiert. Die Analyse und Auswertung der Daten orientierte sich an dem von Mayring (2015) vorgeschlagenen, allgemeinen Ablaufmodell. Insbesondere wurde die qualitative Inhaltsanalyse und dabei die inhaltliche Strukturierung nach Mayring (2015) angewendet, d. h. die Transkripte wurden zunächst inhaltlich in Bezug auf die Forschungsfragen strukturiert, um dann anhand der Dimensionen der Virtualität Kategorien zu bilden. Darüber hinaus wurden Kategorien identifiziert, in denen über die effektive Gestaltung von avatar-basierten Arbeitsumgebungen gesprochen wurden, um Gestaltungsempfehlungen ableiten zu können.

Basierend auf der inhaltlichen Strukturierung nach Mayring (2015) wurden die Kategorien deduktiv gebildet. Hierbei diente das dreidimensionale Modell von Virtualität nach Kirkman und Mathieu (2005) als Grundlage. Um die Kategorien (d. h. Verwendung von IKT, Informationsgehalts der Medien und Synchronität) zu bilden, wurden zunächst die exakten definitorischen Abgrenzungen der Kategorien sowie das Aufstellen von Ankerbeispielen und Kodierungsregeln vorgenommen, um eine eindeutige Zuordnung der Textstellen zu den Kategorien zu gewährleisten. Anschließend wurden mit Hilfe der Abgrenzungsbestimmungen alle Interviews kategorisiert und jede zu einer Kategorie gehörenden Aussage paraphrasiert. Um die Paraphrasen eindeutig den Kategorien zuordnen zu können, wurden gleiche inhaltliche Paraphrasen bis auf eine besonders repräsentative Paraphrase (die Markierzitate, die wir in dieser Studie berichten) gekürzt.

Die Kategorien der Einzelinterviews und der Fokusgruppendiskussion wurden getrennt voneinander identifiziert, um Vergleiche zwischen den Aussagen der erfahrenen und neuen Nutzer*innen ziehen sowie Gemeinsamkeiten und Unterschiede besser herausarbeiten zu können.

### Ethische Überlegungen

Die Rekrutierungsemails, Aufklärungs- und Einwilligungsformulare und der Studienablauf wurden durch die Ethikkommission des Instituts für Psychologie und Arbeitswissenschaft (IPA) der Technischen Universität Berlin geprüft und als unbedenklich eingestuft (#AWB_OEH_01_210531). Alle Gesprächspartner*innen wurden vor Beginn der Studie über das Ziel der Forschung sowie die Freiwilligkeit der Teilnahme und die Möglichkeit der Datenlöschung schriftlich und mündlich informiert. Das Einverständnis wurde von jedem*jeder Teilnehmer*in schriftlich eingeholt.

## Ergebnisse

Die Ergebnisse aus beiden Studienteilen werden in diesem Anschnitt entlang der drei Dimensionen der Virtualität dargestellt (4.1 Verwendung von Informations- und Kommunikationstechnologien, 4.2 Informationsgehalt der Medien und 4.3 Synchronität). Anhand von Markierzitaten aus den Interviews und der Fokusgruppe werden unterschiedliche, teilweise widersprüchliche Ausprägungen dieser jeweiligen Dimensionen verdeutlicht, in den Kontext der Literatur eingebettet und im darauffolgenden Abschn. 5. im Zusammenhang diskutiert. Die Auswahl der hier wiedergegeben Zitate erfolgte mit Fokus auf die Darstellung der Nutzungspraxis in der Zusammenarbeit über avatar-basierte virtuelle Büroumgebungen. Dabei ist vor allem relevant zu zeigen, welche Aspekte von den Studienteilnehmer*innen im Vergleich zu rein virtueller bzw. rein analoger Zusammenarbeit neu gesehen wurden. Es wurden Zitate ausgewählt, die prägnant die jeweiligen Positionen beschreiben und auch kontroverse Haltungen zeigen.

### Verwendung von Informations- und Kommunikationstechnologien

In Bezug auf diese erste Dimension wurden die Antworten der erfahrenen Nutzer*innen auf Fragen nach den Einsatzgründen einer avatar-basierten Büroumgebung sowie deren Einbindung in den Arbeitsalltag ausgewertet. Dabei wurde klar, dass avatar-basierte Arbeitsumgebungen – wie in den vorliegenden Fällen WorkAdventure – als generelle audiovisuelle Kommunikationstechnologie über den gesamten Arbeitstag hinweg verwendet wurde, um interne Abstimmungsprozesse herbeizuführen und Kommunikationsbedarfe zu decken:

„Das [virtuelle Büro] ist einfach wirklich unser HAUPT-Video- und Audiokommunikationstool. […] Jeden Tag. Fünfmal die Woche.“ (Interview E1)

„Also, wenn wir an virtuelle Büros denken, muss man jeden Tag dorthin gehen. Aber wenn ich jeden Tag sage, schließe ich Ereignisse wie Kundentermine oder andere Dinge mit ein. Das ist kein Problem, denn das ist im physischen Büro auch nicht anders. Man ist auch nicht immer von 9 bis 17 Uhr im traditionellen Büro.“ (Interview E5, eigene Übersetzung aus dem Englischen)

Als zweite Funktion der virtuellen Plattform wurde erwähnt, Kolleg*innen aufgabenunabhängig zusammenzuführen und eine dezidierte Plattform für den sozialen Austausch zu kreieren. Neben arbeitsbezogener Kommunikation sollte die avatar-basierte Plattform folglich zur Stärkung des sozialen Gefüges dienen und das Team als Ganzes zusammenführen (Palmer und Speier 1998). Dies wurde aus der Sicht von E1 auch ermöglicht:

„Wir haben aber auch eine Beach-Ecke. Der Strand hat sich so rauskristallisiert, dass wir dort unsere persönlichen Gespräche führen.“ (Interview E1)

Im Falle von E3 wurde zunächst der alltägliche Einsatz von WorkAdventure als vorteilhaft für die Teameffektivität und Motivation wahrgenommen. Über die Zeit wurde die Plattform aber nur noch sporadisch und im Endeffekt gar nicht mehr für sozialen Austausch verwendet:

„Zu Coronazeiten wollten wir das [virtuelle Büro] nutzen, um, naja, so ein Bürofeeling wieder hinzubekommen. Damit die Leute sich auch außerhalb der festgeplanten Meetings treffen. Und auch außerhalb der Teams. […] Also die meisten Leute haben es nicht jeden Tag benutzt, sondern immer nur an ein bis drei Tagen die Woche.“ (Interview E3)

„Am Anfang haben es rund 30 % wirklich genutzt. Punktuell auch manchmal mehr. Dann wurde es immer weniger, irgendwann waren immer die gleichen fünf drinnen, von insgesamt 70 Kolleg*innen – unter 10 %. Daher haben wir es irgendwann eingestellt.“ (Interview E3)

Neben den erfahrenen Nutzer*innen formulierten auch die Teilnehmer*innen der Fokusgruppe, die zum ersten Mal in einer avatar-basierten Büroumgebung agierten, dass die enge Einbindung in den Arbeitsalltag eine zentrale Voraussetzung für die effektive Nutzung der Plattform sei:

„… und wenn man das jetzt noch hinbekommt, dass das standortübergreifend ist, dann wäre das wirklich ein Mehrwert. Dann müsste es aber die Variante sein, dass man den ganzen Arbeitsalltag in so einer Umgebung bringt.“ (Transkript FG)

Die Aussage aus der Fokusgruppendiskussion verdeutlicht, dass die standortunabhängige Zusammenführung von verschiedenen Unternehmensabteilungen nur als umsetzbar wahrgenommen wird, wenn die Plattform als grundlegendes audiovisuelles Kommunikationsmittel verwendet wird. Wenn die Kommunikationszeit über die Plattform steigt, kann dies tatsächlich die effektive Teamzusammenarbeit stärken (Krawczyk-Bryłka 2017), zu einem höheren Informationsaustausch führen (Bhat et al. 2017) und die Frequenz und Dauer formaler Besprechungstermine zu reduzieren:

„Man könnte sich [mit WorkAdventure] wahrscheinlich auch das eine oder andere Meeting sparen, weil man sich einfach einmal vorher kurz eh zufällig gesehen hat: hey, der/die ist im Büro und hat gerade Zeit … Heute macht man eine halbe Stunde Termin in den Kalender, anstatt herüber zu gehen und etwas zu fragen.“ (Transkript FG)

In diesen Ergebnissen zeigt sich deutlich auch für den Kontext avatar-basierter Büroumgebungen, was Griffith und Neale (2001) in Bezug auf den Zusammenhang zwischen Virtualität und der Nutzung von IKT beschrieben haben: Nicht die digitalen Lösungen mit ihren unterschiedlichen Nutzungsmöglichkeiten sind ein entscheidender Faktor für die Virtualität in der Zusammenarbeit, sondern Bandbreite und Häufigkeit der Nutzung im gelebten Arbeitsalltag. Dabei geht es im Falle avatar-basierter virtueller Büroumgebungen weniger um die Nutzung unterschiedlicher Kommunikationslösungen, sondern um die verschiedenen Kontexte innerhalb derer digital mediierte Kommunikation stattfindet: das kann neben dem offiziellen virtuellen Meetingraum für formelle und geplante Absprachen auch der virtuelle Schreibtisch für spontane Nachfragen sein, oder der Strand als Teil der selbstgestalteten Büroumgebung für den sozialen Austausch.

### Informationsgehalt der Medien

In Bezug auf den Einsatz von Medien mit unterschiedlichem Informationsgehalt wurde zunächst der Einsatz der Avatare und die damit verbundenen Ausdrucks- und Interpretationsmöglichkeiten diskutiert. So wurde vor allem in den Einzelinterviews mit den erfahrenen Nutzer*innen deutlich, dass in den individuellen Gestaltungsmöglichkeiten der Avatare eigene Identitätsmerkmale (z. B. durch eine Katze als Begleitung des Avatars) und das aktuelle Befinden (z. B. mit spezifischer Kleidung/Kopfbedeckung) kommuniziert werden können:

„Und manchmal benehme ich mich wie eine Prinzessin, also habe ich eine Krone auf dem Kopf. Weißt du, du musst nicht aussehen wie dein Avatar, aber du kannst deine Persönlichkeit ausdrücken, und das ist wirklich cool. Ich ändere ihn also nicht jeden Tag, damit die Leute dich leicht erkennen können, aber du kannst mit deinem Avatar machen, was du willst. Das ist also auch eine coole Sache.“ (Interview E4)

In der Fokusgruppe mit den Erstnutzer*innen hingegen wurde das Potenzial der Avatare diskutiert, Kommunikationshemmnisse zu durchbrechen, da die Gestaltung der Avatare eine niederschwellige Ebene für ein Einstiegsthema sein könnte. Dabei zeigte sich eine gegenläufige Meinung hinsichtlich der Gestaltung der Avatare in Abhängigkeit der hierarchischen Strukturen.

T1: „Da [bei der Gestaltung des Avatars] kommt es ein bisschen auf das Managementlevel an, was da noch mit herumläuft. Also wenn da unsere Chef Chef Chef mit dabei ist, bin ich wahrscheinlich ein anderer Avatar, als wenn ich nur mit den direkten Kolleg*innen [in WorkAdventure] bin.“

T3: „Obwohl ich gerade dachte, dass durch so etwas ja eigentlich die Hierarchien verschwimmen können, weil man nicht ganz genau weiß; wer ist jetzt eigentlich hier eine Führungskraft? Oder wer ist [aus anderen Teams], eine Ebene höher?“ (Transkript FG)

Durch die Gestaltungsmöglichkeiten der Avatare kann entsprechend der Media-Richness-Theory (Daft und Lengel 1986) demnach persönliche Nuancen wie aktuelle Befindlichkeiten oder die bewusste Abflachung hierarchischer Strukturen mitkommuniziert werden, die sonst primär einer face-to-face Interaktion zugeschrieben werden. Neben der Gestaltung der Avatare als implizite Form der Kommunikation und damit Erweiterung des Informationsgehalts digital mediierter Arbeitsformen spielte auch der Aufenthaltsort der Avatare in der virtuellen Büroumgebung für die Vermittlung impliziter Informationen eine Rolle:

„Es gibt [in unserem virtuellen Büro] verschiedene Bereiche, in denen man sich dementsprechend aufhalten kann, die suggerieren, wie ansprechbar man ist. Das heißt, wenn man [der Avatar] sich in der Essecke aufhält, dann ist klar, dass man gerade eine Pause macht und daher nicht ansprechbar ist. […] Wenn man in der Bibliothek, also unserer Leseecke ist, KANN man gar nicht angesprochen werden. Da ist es auch relativ klar.“ (Interview E1)

Innerhalb der Büroumgebung können die Avatare von den Nutzer*innen in Bereiche bewegt werden, die für die Kolleg*innen eine für die Zusammenarbeit wichtige Information vermittelt. Die implizite Kommunikation von Nichterreichbarkeit durch die Positionierung des eigenen Avatars am Esstisch in der Küche oder in der Bibliothek kann im Umkehrschluss Klarheit in Bezug auf die Ansprechbarkeit für arbeitsrelevante Fragen durch die Positionierung des Avatars am virtuellen Schreibtisch vermitteln. In der Fokusgruppe wurde jedoch angemerkt, dass dies für den gelebten Arbeitsalltag praktikabel umsetzbar sein sollte:

„Man muss auf jeden Fall schauen, dass man es so löst, dass man jetzt nicht genervt ist, dass man seinen Avatar jetzt noch zusätzlich irgendwo hinbewegen muss, wo man selbst gerade, keine Ahnung, vielleicht im [realen] Büro ist und dann in einen anderen Raum [für eine kurze Besprechung] geht“. (Transkript FG)

Innerhalb virtueller Büroumgebungen können demnach individuelle Informationen durch die Gestaltung der Avatare und ihre dynamische Positionierung in der virtuellen Büroumgebung vermittelt werden, die den Informationsgehalt dieses Mediums deutlich umfangreicher gestalten kann als herkömmliche Anwendungen für Online-Meetings.

### Synchronität

Die dritte Dimension der Virtualität bildet sich zwischen den zwei Extremen der synchronen und asynchronen Zusammenarbeit ab (Dennis et al. 2008; Kirkman und Mathieu 2005). Die avatar-basierten Plattformen wurden von den erfahrenen Nutzer*innen als vorwiegend synchrone Arbeitsumgebung beschrieben, die nur funktioniere, wenn jedes Teammitglied bereit ist, gleichzeitig im virtuellen Büro zu sein.

„Ich würde sagen, dass die Leute alle gleichzeitig arbeiten, weil man bei WorkAdventure nicht asynchron sein kann. Man muss zur gleichen Zeit auf der Plattform sein, um mit den Leuten reden zu können.“ (Interview E4)

Avatar-basierte Arbeitsumgebungen sind eine Weiterentwicklung der bereits etablierten Videokonferenztools. Der wesentliche Unterschied zur herkömmlichen Videokonferenzsoftware ist jedoch, dass der virtuelle Raum auch dann existiert, wenn kein Dialog stattfindet. So können Personen flexibel entscheiden, wann sie den Raum betreten, wie viel sie kommunizieren und ob ihre Kommunikationsweise der synchronen Form bedarf. Innerhalb dieser vornehmlich synchrone Arbeitsformen unterstützenden virtuellen Büroumgebung gibt es jedoch noch feinere Abstufungen. Die erfahrenen Nutzer*innen beschrieben eine situations- und aufgabenbezogene Nutzung der Kommunikationsoptionen in der virtuellen Büroumgebung, also ob sie ihren Avatar auf den eines*r Kollegin zusteuerten und damit einen ad-hoc Videocall initialisierten, oder die Chatfunktion innerhalb der Plattform nutzten. Die Videocall-Funktion wurde dabei vermehrt als eine schnelle, unkomplizierte Kommunikationsmöglichkeit angesehen, um komplexere Thematiken zu klären und Nachfragen zu stellen (Afflerbach 2020; Bell und Kozlowski 2002). Der Chat diente dahingegen eher dem Zweck, Sachverhalte, die keinen unmittelbaren Klärungsbedarf hatten, auszutauschen und die Arbeit anderer nicht ständig zu unterbrechen.

„Zum Beispiel bei einem Statusupdate, wo du einfach noch gar nicht weißt, was meine nächste Frage ist, oder du je nachdem nicht weißt welche Antworten kommen, wenn es noch so ein bisschen diffus ist, wie das Gespräch laufen kann. Dann ist einfach aufeinander zugehen, also so ein [spontaner] Videochat das wichtigste“. (Interview E1)

„Ich denke, man kann WorkAdventure nicht wie einen Chat benutzen. Denn wenn man zu jemandem geht, wird man diese Person verärgern, man wird die Person unterbrechen. Man muss also etwas fragen, das für die weitere Arbeit wichtig ist. Aber wenn man ein paar Minuten auf eine Antwort warten kann, ist es besser, die Chatfunktion zu benutzen, weil man die Leute nicht stört und sie sich ihre Zeit besser einteilen können. Sie können also selbst entscheiden, wann sie im Chat antworten wollen und so weiter. Ich denke also, man muss sich überlegen, wie man diese verschiedenen Kanäle nutzt, um nicht zu sehr zu nerven.“ (Interview E4)

Avatar-basierte Arbeitsumgebungen stellen demnach ein Kommunikationsmittel dar, welches den persönlichen, synchronen Austausch anregt: Feedback, komplexeres Problemlösen und Abstimmungen können in vereinfachter, schnellerer Form stattfinden und fördern somit die generelle Teameffektivität. Es gibt eine Chatfunktion, die allerdings nur für den unmittelbaren Austausch genutzt und nicht gespeichert werden kann. Ist die Niederschrift von Information und der spätere Abruf von Informationen notwendig, muss neben den avatar-basierten Plattformen eine weitere textuelle Softwareanwendung verwendet werden:

„Im Moment ist es nicht möglich, z. B. ein Post-It auf dem Schreibtisch [der virtuellen Büroumgebung] zu hinterlassen, damit es später jemand sieht. Es ist also nicht für eine asynchrone Nutzung geeignet.“ (Interview E4)

„Und für mich ist das schon so, dass ich viele Tasks dort [in meiner Kalender-App] notiere, dann aber sehr häufig eben im WorkAdventure kurz bei dem jeweiligen vorbeigehe und frage: „Hey, woran bist du gerade? Brauchst du da Input?“, und so weiter.“ (Interview E2)

Für asynchrone Modi der Zusammenarbeit haben die erfahrenen Nutzer*innen demnach neben der Nutzung der Chatfunktion in bestimmten Situationen Lösungen im Zusammenspiel mit anderen Anwendungen gefunden. Dabei wird hier der Bedarf nach weiteren Lösungen für die Einbindung asynchronener Zusammenarbeit in einer avatar-basierten Plattform klar formuliert, um eine zeitlich unabhängige Zusammenarbeit zu fördern.

## Diskussion

### Virtualität in avatar-basierten Büroumgebungen

Die vorliegenden Ergebnisse zeigen die Komplexität hybrider Zusammenarbeit anhand der Perspektiven von erfahrenen und neuen Nutzer*innen avatar-basierter virtueller Büroumgebungen. Dabei beeinflussen mehrere Dimensionen die von den Nutzer*innen wahrgenommenen Vorteile und Herausforderungen dieser virtuellen Plattformen für den Arbeitsalltag. Mit der Betrachtung von Virtualität als mehrdimensionales Konstrukt zeigen die Ergebnisse der Studie in Bezug auf die **Verwendung von IKT**, dass es in avatar-basierten Arbeitsumgebungen weniger um die Unterscheidung verschiedener IKT-Lösungen geht, sondern um den Nutzungskontext: Digital mediierte Kommunikation findet innerhalb der virtuellen Büroumgebung im offiziellen virtuellen Meetingraum für formelle und geplante Absprachen statt, aber auch am virtuellen Schreibtisch für spontane Nachfragen oder am digitalen Strand für sozialen Austausch. Dabei ist hier die genutzte IKT immer die Video-Call-Funktion, die in die virtuelle Büroumgebung integriert ist. Jedoch definiert der virtuelle Kontext, innerhalb derer die Interaktion stattfindet, den Rahmen für Motivation, Zweck und Ausgestaltung der jeweiligen Zusammenarbeit. Während der Nutzungskontext für die Gestaltung von IKT allgemein von Relevanz ist (Harrison et al. 2007), stellen die in den Ergebnissen skizzierten Perspektiven der Nutzer*innen auf die virtuellen Nutzungskontexte eine spezifische Erweiterung für Verwendung von IKT in virtuellen Arbeitsumgebungen dar.

Bei der Betrachtung des** Informationsgehalts der Medien** zeigen die Ergebnisse in Bezug auf die Gestaltungsmöglichkeiten des Avatars und der Bedeutungszuschreibungen verschiedener Orte innerhalb der virtuellen Büroumgebung, dass dadurch eine höhere Virtualität erreicht werden kann, die wiederum essenziell für eine erfolgreiche Zusammenarbeit ist. Denn die Sichtbarkeit der Position der Avatare von Kolleg*innen an unterschiedlichen Orten im Büro zeigt an, ob ein spontanes Ansprechen möglich ist oder nicht: Sitzt ein Avatar am Schreibtisch, so ist nach den Aussagen unserer Studienteilnehmer*innen klar, dass die Person weder in einem Meeting ist noch sich in einem direkten Gespräch mit einer Person befindet. Die Positionierung in der Bibliothek macht dagegen deutlich, dass keine Störung gewünscht ist. Ein Avatar in der Kaffeeküche zeigt an, dass eine Person gerade in der Pause und vermutlich nicht erreichbar ist, oder – je nach Gepflogenheiten im Team – gerade jetzt für einen informellen Plausch verfügbar. Damit ergibt sich hier eine zusätzliche Informationsebene, die eine Indikation für die aktuelle Arbeitssituation der Kolleg*innen vermittelt und damit spontane, kurzfristige virtuelle Interaktion im Sinne des typischen Flurgesprächs im physischen Büro möglich macht.

Bestimmte Gestaltungsoptionen des Avatars, wie hier im Interview durch das Aufsetzen einer Krone, können zudem Rückschlüsse auf die aktuelle Befindlichkeit der einzelnen Personen zulassen, jedoch mit viel Raum für (Fehl‑)Interpretation. Um das Potenzial des gesteigerten Informationsgehalts in der Arbeitspraxis nutzen zu können, so deuten die Ergebnisse an, ist es wichtig, die (mehr oder weniger) expliziten Bedeutungszuschreibungen für alle Kolleg*innen in der virtuellen Büroumgebung einheitlich zu klären und Regeln festzulegen, um Missverständnisse und Ineffizienzen zu vermeiden. Denn es kann nur über einen Team-internen Konsens geklärt werden, ob die Positionierung des Avatars an einem virtuellen Esstisch in der Küche die Nichterreichbarkeit oder im Gegenteil eine Einladung zur Interaktion signalisiert.

In Bezug auf die **Synchronität** als dritte Dimension von Virtualität liefern die Ergebnisse klare Anhaltspunkte dafür, dass die in der Literatur als dichotome Ausprägungen charakterisierten Pole von synchroner und asynchroner Zusammenarbeit (Kirkman und Mathieu 2005) in avatar-basierten virtuellen Büroumgebungen dichter ineinandergreifen: Wenngleich als primär synchrones Arbeitsmedium zur Erlangung von kommunikativer Konvergenz (Dennis et al. 2008) durch die Einbindung der Video-Call Funktion konzipiert, wurde die im avatar-basierten System eingebaute Chatfunktion auch für zumindest kurzfristig asynchrone Modi der Zusammenarbeit eingesetzt, etwa um eine weniger zeitkritische Frage zu stellen. Für eine nachhaltige Speicherung von Aufgabenlisten musste jedoch eine weitere Lösung herangezogen werden. Damit wird das Zusammenspiel synchroner und asynchroner Arbeitsmodi in der gelebten Arbeitspraxis der erfahrenen Nutzer*innen als ein dynamisches und eng verzahntes Zusammenspiel beschrieben und weniger als eine klare Trennung zweier gegensätzlicher Pole.

Mit den avatar-basierten Plattformen können Teams folglich räumliche Distanzen überbrücken und eine an die dynamisch wechselnden Bedingungen des tatsächlich gelebten Arbeitsalltags angepasste Kommunikation umsetzen, auch wenn die Zusammenarbeit am selben Ort eingeschränkt ist. Diese virtuellen Räume ermöglichen es, einen höheren Grad an nonverbalen Informationen zu übermitteln als mit einem Gefüge unterschiedlicher Tools zur Kommunikation und dadurch mehr Spontanität in die virtuelle Zusammenarbeit zu bringen. Ähnliche Effekte konnte auch für die Lehre in der Hochschule im Rahmen einer Studie gezeigt werden, bei der die Hälfte der Student*innen mit Zoom unterrichtet wurden, die andere Hälfte ihren Kurs in der virtuellen Büroumgebung gather.town absolvierte. Hier zeigten die Student*innen eine klare Präferenz für die Teilnahme am Kurs über gather.town (Latulipe und De Jaeger 2022).

### Praxisrelevanz

Aus den Ergebnissen lassen sich für den Einsatz in der Praxis drei zentrale Bedingungen herausarbeiten:

1. Virtuelle Büroumgebung als Spiegel des Arbeitsalltags
  Eine zentrale Bedingung für das Ausschöpfen des Potenzials avatar-basierter virtueller Büroumgebungen in hybrid arbeitenden Teams ist, dass die virtuelle Plattform als alltägliches Tool genutzt wird. Werden die Lösungen primär als interessante Alternative für digitale Besprechungen eingesetzt, kann das Potenzial eines erhöhten Informationsgehalts nicht genutzt werden. Der intensiven Nutzung steht insofern eine Herausforderung entgegen, wenn nutzende Personen mehreren Teams zugehörig sind und somit umfangreichere Büroumgebungen notwendig machen. Diesen Fall unterstützen die existierenden Plattformen bisher nur eingeschränkt, da für mehrere Teams größere Arbeitsumgebungen notwendig sind, zu große Umgebungen aber zu übermäßig langen Wegen in der virtuellen Welt und damit Ineffizienzen führen.
2. Aussagekräftiger Aufbau der Büroumgebung
  Das virtuelle Büro sollte klar erkennbare (bzw. definierbare) unterschiedliche Bereiche haben, wie etwa einen Bereich mit Schreibtischen, Besprechungsräume oder Konversationsbereiche wie eine Sitzecke oder Kaffeeküche sowie definierte „Rückzugsorte“ wie eine Bibliothek. Zudem sollten klare Regeln festgelegt werden, in welchen Umgebungen welche Art von Interaktionen gewünscht und angebracht sind. Damit können die Avatare in den entsprechenden Bereichen positioniert werden und eine erweiterte Statusinformation übermitteln. Nach der Ansicht der Studienteilnehmer*innen kann dies spontane „Flurgespräche“ ermöglichen und so einige formale Besprechungen ersetzen.
3. Interaktionsmodi vorab klären
  Flurgespräch oder Statusupdate? Um die Vorteile des informellen Charakters spontaner virtueller Flurgespräche nutzen zu können, sollten umgekehrt die Kontexte für formellere Interaktionen geklärt sein, etwa durch das Markieren spezifischer Orte innerhalb des virtuellen Büros sowie die Bereitstellung dafür notwendiger IKT, die je nach Bedarf Abstufungen von synchroner bzw. asynchroner Kommunikation (z. B. Chats mit Speicher- und Löschfunktion) unterstützen.

### Kritische Würdigung und Forschungsausblick

Diese Studie ermöglicht eine Erstbetrachtung der Nutzung avatar-basierter virtueller Büroumgebungen anhand einer kleinen und spezifisch ausgewählten Stichprobe. Damit sind der Breite, in der die hier gezeigten Ergebnisse auf andere Kontexte übertragbar sind, klare Grenzen gesetzt. Jedoch ist der Einsatz avatar-basierter Lösungen im tatsächlich gelebten Arbeitsalltag von Organisationen ein neues Phänomen. Bisherige Studien haben überwiegend über die Nutzung avatar-basierter Büroumgebungen im Rahmen von Lehr‑/Studienprojekten oder Online-Konferenzen berichtet (Honda et al. 1997; Latulipe und De Jaeger 2022; Latulipe und Turnbull 2022; Najjar et al. 2022; Silva et al. 2022). Breitere empirische Befunde über die Ausprägung in sozio-technischen Arbeitssystemen stehen noch aus.

Die hier vorliegende Studie ist ein erster Schritt in diese Richtung und bildet einen Ausgangspunkt für zukünftige Forschung über die Nutzung avatar-basierter Arbeitsumgebungen in Unternehmen und Teams sowie empirische Effizienzbetrachtungen. Für eine Erweiterung des theoretischen Beitrags wäre zukünftige Forschung insbesondere in Bezug auf die Dimension Synchronität im Kontext von Virtualität vielversprechend, da avatar-basierte Arbeitsumgebungen hier bisher Defizite aufweisen. Zudem wäre eine Erhebung der konkreten Tätigkeiten und Arbeitsabläufe zwischen einzelnen Teammitgliedern wünschenswert, wofür aktuell jedoch noch die methodischen Erhebungsinstrumente genauer auf die Anforderungen hinsichtlich Anonymität und Teilnehmer*innenschutz kalibriert werden müssen. Mit einer Erweiterung der methodischen Basis auf virtuelle in-situ Beobachtung bzw. virtuelle Ethnografie (Gupper und Mörike 2022) könnten somit detailgenauere Einblicke in die gelebte Arbeitspraxis erlangt werden, um ein mehrschichtiges Bild vom Zusammenspiel synchroner und asynchroner Arbeitsmodi herauszuarbeiten.

## Conclusio

Im Hinblick auf die Forschungsfragen dieses Beitrags, (1) wie der Arbeitsalltag und die Zusammenarbeit in diesen Umgebungen koordiniert wird und (2) welche Vorteile und Herausforderungen erfahrene und neue Nutzer*innen in Bezug auf diese Arbeitsform sehen (2), lässt sich festhalten, dass diese virtuellen Plattformen den Anfang von vielen kreativen Lösungen im Bereich der virtuellen, hybriden Zusammenarbeit darstellen. Für die nachhaltig erfolgreiche Nutzung avatar-basierter Büroumgebungen hat sich jedoch als wichtigste Voraussetzung gezeigt, dass die Teammitglieder bereit sein müssen, die Plattform für die meisten internen Abstimmungsprozesse regelmäßig zu verwenden, da sonst die erweiterte Vermittlung persönlicher Nuancen in der Kommunikation sowie implizite Informationen zur Verfügbarkeit einzelner Teammitglieder nicht über konsensual ausgehandelte Bedeutungszuschreibungen zugänglich werden. Erst die Entstehung eines komplexeren Gefüges aus spezifischen Orten innerhalb der Plattform, Gestaltung und Positionierung der Avatare und dem dynamischen Zusammenspiel synchroner und asynchroner Arbeitsmodi machen avatar-basierte virtuelle Büroumgebungen zu mehr als einer Plattform für Video-basierte Interaktion. Sind diese Voraussetzungen gegeben, können darüber hinaus zusätzliche Informationen vermittelt werden, die zum einen virtuelle Flurgespräche ermöglichen und damit einige der formellen Besprechungen ersetzen, zum anderen Räume für unterschiedliche Gesprächsmodi vom Teeküchen-Plausch bis zum Status-Update eröffnen.

## Anhang

### Leitfaden für halbstrukturierte Einzelinterviews

**Table 2:**

**Leitfrage/Stimuli/Erzählaufforderung**		
Wofür wird in deinem Unternehmen die avatarbasierte Arbeitsumgebung genutzt?		
**Inhaltliche Aspekte**	**Aufrechterhaltungsfragen**	**Nachfragen**
*Allgemeine Einschätzung der virtuellen Zusammenarbeit über WorkAdventure*	Wie kann ich mir die Benutzung bei dir genau vorstellen?	Wie bist du zu der avatarbasierten Arbeitsumgebung gekommen? ODER Was ist die Idee hinter der avatarbasierte Arbeitsumgebung? Wie häufig wird es von dir in der Woche genutzt? Welche verschiedenen avatarbasierten Arbeitsumgebungen kennst du?
**Leitfrage/Stimuli/Erzählaufforderung**		
Würdest du mir bitte deine Arbeitsumgebung einmal vorstellen und mich darin rumführen?		
**Inhaltliche Aspekte**	**Aufrechterhaltungsfragen**	**Nachfragen**
*Gestaltung der WorkAdventure-Umgebung*	–	Welche unterschiedliche Räume gibt es? Wofür werden die Räume benutzt? Welche Funktionen können integriert werden?
**Leitfrage/Stimuli/Erzählaufforderung**		
Für welchen Zweck verwendest du die unterschiedliche Kontaktwege?		
**Inhaltliche Aspekte**	**Aufrechterhaltungsfragen**	**Nachfragen**
*Verwendung von IKT’s Kommunikation*	Wann verwendest du bspw. Emails/Chats/Telefon/persönliches Gespräch/Videokonferenzen?? Welche Kontaktwege sind davon in deiner avatarbasierten Arbeitsumgebung beinhaltet? ODER Welche Kontaktwege können in die avatarbasierte Arbeitsumgebung integriert werden?	Auf welche Weise hat die avatarbasierte Arbeitsumgebung die Kommunikationshäufigkeit über andere Tools beeinflusst? Wie häufig trefft ihr euch noch zu einem Kaffee im Team und redet über arbeitsunabhängige Themen während der Arbeit? Wie hat die avatarbasierte Arbeitsumgebung das beeinflusst? ODER Wie kann die avatarbasierte Arbeitsumgebung das beeinflussen?
**Leitfrage/Stimuli/Erzählaufforderung**		
Wie häufig arbeitest du zeitgleich miteinander zusammen?		
**Inhaltliche Aspekte**	**Aufrechterhaltungsfragen**	**Nachfragen**
*Synchronität*	Hat die avatarbasierte Arbeitsumgebung die Zeit, in der gleichzeitig gearbeitet wird, verändert? Warum? Warum nicht? ODER Inwieweit kann die avatarbasierte Arbeitsumgebung zu einer synchronen Arbeitsweise im Homeoffice führen?	Wie häufig bearbeitest du deine Aufgaben alleine oder zusammen mit deinen Teammitgliedern? Geschieht das zeitgleich?
